# Application of Dynamic Analysis in Semi-Analytical Finite Element Method

**DOI:** 10.3390/ma10091010

**Published:** 2017-08-30

**Authors:** Pengfei Liu, Qinyan Xing, Dawei Wang, Markus Oeser

**Affiliations:** 1 Institute of Highway Engineering, RWTH Aachen University, Mies-van-der-Rohe-Street 1, D52074 Aachen, Germany; liu@isac.rwth-aachen.de (P.L.); oeser@isac.rwth-aachen.de (M.O.); 2 Department of Civil Engineering, Tsinghua University, Beijing 100084, China; xingqy@tsinghua.edu.cn; 3 Institute of Highway Engineering, Paul-Bonatz-Street 9–11, University of Siegen, D57076 Siegen, Germany

**Keywords:** dynamic analysis, semi-analytical finite element method, asphalt pavement, moving loads, pavement design and diagnostics

## Abstract

Analyses of dynamic responses are significantly important for the design, maintenance and rehabilitation of asphalt pavement. In order to evaluate the dynamic responses of asphalt pavement under moving loads, a specific computational program, SAFEM, was developed based on a semi-analytical finite element method. This method is three-dimensional and only requires a two-dimensional FE discretization by incorporating Fourier series in the third dimension. In this paper, the algorithm to apply the dynamic analysis to SAFEM was introduced in detail. Asphalt pavement models under moving loads were built in the SAFEM and commercial finite element software ABAQUS to verify the accuracy and efficiency of the SAFEM. The verification shows that the computational accuracy of SAFEM is high enough and its computational time is much shorter than ABAQUS. Moreover, experimental verification was carried out and the prediction derived from SAFEM is consistent with the measurement. Therefore, the SAFEM is feasible to reliably predict the dynamic response of asphalt pavement under moving loads, thus proving beneficial to road administration in assessing the pavement’s state.

## 1. Introduction

The analysis of loading in finite element (FE) simulation of asphalt pavement can be modelled in two types: dynamic or static analyses. Static analysis is relatively simpler and thus reduces computational time. This loading type has advantages when elastic materials are investigated due to the exactly same loading and unloading path for elastic materials [1]. The early studies by Duncan et al. [2], Raad and Figueroa [3] and Harichandran et al. [4] applied static analysis on asphalt concrete surface, which were followed by more researchers using static analysis to investigate other aspects of asphalt pavement [5,6,7]. However, the inertial forces and time dependency of materials cannot be taken account in the static analysis, because it ignores the force induced by mass and material damping, which is not the reality of the pavement.

Zaghloul and White [8] conducted one of the first studies of dynamic analysis in the general-purpose finite element method (FEM) program ABAQUS in the year of 1993, in which uniformly distributed pressure varying as a trapezoidal shape in time was used to model a moving load. In this study, it was found that when the speed of loading increased, the surface deflection decreased and there was more deflection derived from static loading than from dynamic loading. A dynamic analysis of flexible pavement was carried out by Desai and Whitenack [9], which was further developed by Desai [10]. The series of studies proposed a new material constitutive model to model the distress induced in pavement during dynamic loading. The stored strain energy was used to consider the effect of material changes caused by cycles of loading. Particularly, a reduced dissipation of energy happened in each cycle, leading to stiffer material behavior in the following cycle. Saad et al. [11] assumed the dynamic loading as a triangular pulse in a 0.1 s period and then performed a dynamic analysis of flexible pavement. The researchers pointed out that the results of surface deflection calculated from dynamic loading may be less than half of the value derived from static loading. The reason was believed to be that the absorption of energy by the damping and mass inertia of the pavement system. A comprehensive review of the previous methods to simulate dynamic loading was made by Beskou and Theodorakopoulos [12]. They categorized the dynamic analysis according to representative models for asphalt pavement. The dynamic analysis of a pavement system can be achieved by analytical method, hybrid method of analytical and numerical methods or purely numerical method. Only numerical methods can solve realistic problems with complex material behavior and geometries; the FEM, the boundary element method (BEM) and the BEM/FEM hybrid scheme are the most popular numerical methods. Considering efficiency, availability, versatility and accuracy, the FEM appears to be the best. Most of the layered pavement systems were analyzed by linear formulation with respect to the material behavior and deformation. The dynamic analysis of these models required the development of an innovative efficient and accurate numerical method and its implementation in computer software.

One method was proposed to meet the requirement of accuracy and efficiency, which is the so-called semi-analytical finite element method (SAFEM) [13,14,15,16,17,18,19,20]. This method was first developed by Wilson [21] for linear analysis and Meissner [22] extended Wilson’s work to an elastoplastic body. Winnicki and Zienkiewicz [23] adopted a viscoplastic formulation to deal with material nonlinearity. An efficient analysis of the consolidation of elastic bodies’ under nonsymmetric loading was provided by Carter and Booker [24] through continuous Fourier series. A discrete Fourier technique was successfully applied by Lai and Booker [25] to analyze the nonlinear behavior of solids under three-dimensional (3D) loading conditions. Fritz [13] and Hu et al. [14] further developed the SAFEM, and programed specific FE codes using this method to analyze the problems of asphalt pavement. However, their FE codes are relatively simple, e.g., only static analysis with linear elastic material properties and a total bond between pavement layers can be performed. As a result, the dynamic analysis should be further applied to the SAFEM. In the following sections, the mathematical basis of the SAFEM and the algorithm of application of the dynamic analysis to SAFEM will be introduced, followed by the verification using the results derived from commercial FE software (ABAQUS) and the field measurement. A brief summary and conclusions are provided at the end.

## 2. Description of Semi-Analytical Finite Element Method

**Figure 1:**
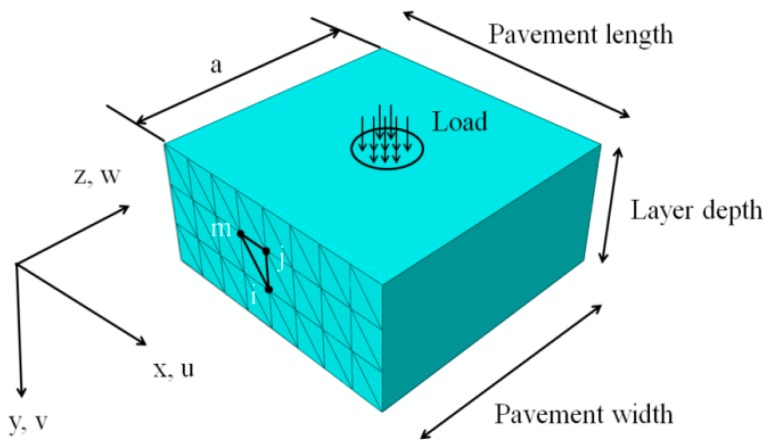
Schematic representation of an SAFEM situation.

For the sake of simplification, the FE solution for static analysis is introduced first. The shape functions in a 3D SAFEM model, which are to define the variation of displacements, are written as a two-dimensional (2D) traditional shape function multiplied by Fourier series, as shown in Figure 1:

(1) $$N_{k}\left(x,y,z\right)=\left[\bar{N_{k}}\left(x,y\right)cos\frac{l\pi z}{a}\right]+\left[\overset{=}{N_{k}}\left(x,y\right)sin\frac{l\pi z}{a}\right]$$

where *l* identifies the term of the Fourier series and is up to *L*, which is the number of total terms; $\bar{N_{k}}$ and $\overset{=}{N_{k}}$ are the shape functions of the node in the XY plane.

In order to derive the displacements in three dimensions, three degrees of freedom at each node of the triangular finite element should be considered. The vector of nodal displacements is:

(2) $$d_{k}=\left\{\begin{array}{c} u_{k}\\ v_{k}\\ w_{k} \end{array}\right\},\;k=1,\;2,\;3\ldots 6$$

where $u_{k}$, $v_{k}$ and $w_{k}$ are the displacements of the node k in *x*-, *y*- and *z*-directions, respectively.

Displacements at some point inside the element can be determined with the use of nodal displacements $\left\{d_{k}\right\}$ and shape functions $N_{k}$:

(3) $$d=\overset{6}{\underset{k=1}{\sum }}N_{k}\left(x,y,z\right)d_{k}=\overset{L}{\underset{l=1}{\sum }}\overset{6}{\underset{k=1}{\sum }}\left\{\left[\bar{N_{k}}\left(x,y\right)cos\frac{l\pi z}{a}\right]+\left[\overset{=}{N_{k}}\left(x,y\right)sin\frac{l\pi z}{a}\right]\right\}d_{k}^{l}$$

The loading function defining the variation of load along the *z*-direction is given by [15]:

(4) $$f=\overset{L}{\underset{l=1}{\sum }}\left\{\left[\bar{p}\left(x,y\right)cos\frac{l\pi z}{a}\right]+\left[\overset{=}{p}\left(x,y\right)sin\frac{l\pi z}{a}\right]\right\}$$

where $\bar{p}\left(x,y\right)$ and $\overset{=}{p}\left(x,y\right)$ represent the pavement load.

The pavement is assumed to be supported at both edges (*z* = 0 and *z* = *a*), particularly, all displacements in the XY plane are prevented, while the motion in the *z*-direction is unrestricted. In order to meet this requirement, the displacement functions with three components *u*, *v* and *w* are rewritten as follows:

(5) $$\begin{array}{c} d=\{\begin{array}{c} u\\ v\\ w \end{array}\}=\sum_{l=1}^{L}\sum_{k=1}^{6}N_{k}\left[\begin{array}{ccc} sin\frac{l\pi z}{a} & 0 & 0\\ 0 & sin\frac{l\pi z}{a} & 0\\ 0 & 0 & cos\frac{l\pi z}{a} \end{array}\right]\{\begin{array}{c} u_{k}^{l}\\ v_{k}^{l}\\ w_{k}^{l} \end{array}\}\;=\sum_{l=1}^{L}N^{l}\cdot d^{l}\\ \mathrm{with}\;N^{l}=N_{k}\left[\begin{array}{ccc} \sin\frac{l\pi z}{a} & 0 & 0\\ 0 & \sin\frac{l\pi z}{a} & 0\\ 0 & 0 & \cos\frac{l\pi z}{a} \end{array}\right] \end{array}$$

Similarly, the function of loading can be formulated as [13]:

(6) $$\begin{array}{c} f=\sum_{l=1}^{L}p\left(x,y\right)sin\frac{l\pi z}{a}=\sum_{l=1}^{L}{\left\{p\right\}}^{l}\\ \mathrm{with}\;p\left(x,y\right)=\overset{n}{\underset{t=1}{\sum }}\left(\frac{2P_{t}}{l\pi }\right)\left[cos\frac{l\pi }{a}Z_{t1}-cos\frac{l\pi }{a}Z_{t2}\right] \end{array}$$

where *P_t_* is the tire load pressure; *Z_t_*_1_ is the *z* coordinate where the tire load area starts; *Z_t_*_2_ is the *z* coordinate where the tire load area ends.

Through displacements at nodal points the strains are determined as:

(7) $$\begin{array}{c} \varepsilon =\{\begin{array}{c} \varepsilon_{x}\\ \varepsilon_{y}\\ \varepsilon_{z}\\ \gamma_{xy}\\ \gamma_{yz}\\ \gamma_{zx} \end{array}\}=\;\sum_{l=1}^{L}\{\begin{array}{c} \frac{\partial u^{l}}{\partial x}\\ \frac{\partial v^{l}}{\partial y}\\ \frac{\partial w^{l}}{\partial z}\\ \frac{\partial u^{l}}{\partial y}+\frac{\partial v^{l}}{\partial x}\\ \frac{\partial v^{l}}{\partial z}+\frac{\partial w^{l}}{\partial y}\\ \frac{\partial u^{l}}{\partial z}+\frac{\partial w^{l}}{\partial x} \end{array}\}\\ =\sum_{l=1}^{L}\sum_{k=1}^{6}\left[\begin{array}{ccc} \frac{\partial N_{k}}{\partial x}u_{k}^{l}\sin\frac{l\pi z}{a} & 0 & 0\\ 0 & \frac{\partial N_{k}}{\partial y}v_{k}^{l}\sin\frac{l\pi z}{a} & 0\\ 0 & 0 & -\frac{l\pi }{a}N_{k}w_{k}^{l}\sin\frac{l\pi z}{a}\\ \frac{\partial N_{k}}{\partial y}u_{k}^{l}\sin\frac{l\pi z}{a} & \frac{\partial N_{k}}{\partial x}v_{k}^{l}\sin\frac{l\pi z}{a} & 0\\ 0 & \frac{l\pi }{a}N_{k}v_{k}^{l}\cos\frac{l\pi z}{a} & \frac{\partial N_{k}}{\partial y}w_{k}^{l}\cos\frac{l\pi z}{a}\\ \frac{l\pi }{a}N_{k}u_{k}^{l}\cos\frac{l\pi z}{a} & 0 & \frac{\partial N_{k}}{\partial x}w_{k}^{l}\cos\frac{l\pi z}{a} \end{array}\right]\\ =\;\sum_{l=1}^{L}\sum_{k=1}^{6}\left[\begin{array}{ccc} \frac{\partial N_{k}}{\partial x}\sin\frac{l\pi z}{a} & 0 & 0\\ 0 & \frac{\partial N_{k}}{\partial y}\sin\frac{l\pi z}{a} & 0\\ 0 & 0 & -\frac{l\pi }{a}N_{k}\sin\frac{l\pi z}{a}\\ \frac{\partial N_{k}}{\partial y}\sin\frac{l\pi z}{a} & \frac{\partial N_{k}}{\partial x}\sin\frac{l\pi z}{a} & 0\\ 0 & \frac{l\pi }{a}N_{k}\cos\frac{l\pi z}{a} & \frac{\partial N_{k}}{\partial y}\cos\frac{l\pi z}{a}\\ \frac{l\pi }{a}N_{k}\cos\frac{l\pi z}{a} & 0 & \frac{\partial N_{k}}{\partial x}\cos\frac{l\pi z}{a} \end{array}\right]\left\{\begin{array}{c} u_{k}^{l}\\ v_{k}^{l}\\ w_{k}^{l} \end{array}\right\}\\ =\;\sum_{l=1}^{L}\sum_{k=1}^{6}B_{k}^{l}d_{k}^{l}=\;\sum_{l=1}^{L}B^{l}\cdot d^{l}\\ \mathrm{with}\;B^{l}=\;[B_{1}^{l},B_{2}^{l},\ldots ,B_{k}^{l}] \end{array}$$

Matrix B is called the strain-displacement matrix. $B_{k}^{l}$ is the strain-displacement matrix of the node *k* at *l*th term of the Fourier series. It is obtained by differentiation of displacements in the form of shape functions and nodal displacements:

(8) $$B_{k}^{l}=\left[\begin{array}{ccc} \frac{\partial N_{k}}{\partial x}sin\frac{l\pi z}{a} & 0 & 0\\ 0 & \frac{\partial N_{k}}{\partial y}sin\frac{l\pi z}{a} & 0\\ 0 & 0 & -\frac{l\pi }{a}N_{k}sin\frac{l\pi z}{a}\\ \frac{\partial N_{k}}{\partial y}sin\frac{l\pi z}{a} & \frac{\partial N_{k}}{\partial x}sin\frac{l\pi z}{a} & 0\\ 0 & \frac{l\pi }{a}N_{k}cos\frac{l\pi z}{a} & \frac{\partial N_{k}}{\partial y}cos\frac{l\pi z}{a}\\ \frac{l\pi }{a}N_{k}cos\frac{l\pi z}{a} & 0 & \frac{\partial N_{k}}{\partial x}cos\frac{l\pi z}{a} \end{array}\right]=B1_{k}^{l}sin\frac{l\pi z}{a}+B2_{k}^{l}cos\frac{l\pi z}{a}$$

The $B_{k}^{l}$ in Equation (8) is split into two matrices—each includes only one set of trigonometric terms.

Now, the general form of total potential energy can be expressed through nodal displacements:

(9) $$\Pi \left(d\right)=\int_{V}^{}\frac{1}{2}{\left(\left[B\right]\left\{d\right\}\right)}^{T}\left[D\right]\left(\left[B\right]\left\{d\right\}\right)dV-\int_{V}^{}{\left(\left[N\right]\left\{d\right\}\right)}^{T}\left\{b\right\}dV-\int_{S}^{}{\left(\left[N\right]\left\{d\right\}\right)}^{T}\left\{f\right\}dS$$

Nodal displacements {d} corresponding to the minimum of the functional Π are determined by the following condition:

(10) $$\left\{\frac{\partial \Pi }{\partial d}\right\}=0$$

The following equilibrium equation is produced by differentiation of Π in respect to {d} for one element:

(11) $$\int_{V}^{}{\left[B\right]}^{T}\left[D\right]\left[B\right]dV\left\{d\right\}-\int_{V}^{}{\left[N\right]}^{T}\left\{b\right\}dV-\int_{S}^{}{\left[N\right]}^{T}\left\{f\right\}dS\;=0$$

which can be reformulated as follows:

(12) $$\begin{array}{c} \left[k\right]\left\{d\right\}=\left\{f\right\}\\ \mathrm{with}\;\left[k\right]=\int_{V}^{}{\left[B\right]}^{T}\left[D\right]\left[B\right]dV\;\mathrm{and}\;\left\{f\right\}=\int_{V}^{}{\left[N\right]}^{T}\left\{b\right\}dV+\int_{S}^{}{\left[N\right]}^{T}\left\{f\right\}dS \end{array}$$

Here [*k*] is the stiffness matrix of one element; {f} is the vector of load.

From Equations (8) and (12), the stiffness matrix of one element includes the following integrals due to [*B*] [14]:

(13) $$\begin{array}{c} I_{1}=\int_{0}^{a}sin\frac{l\pi z}{a}\cdot cos\frac{m\pi z}{a}\cdot dz\\ I_{2}=\int_{0}^{a}sin\frac{l\pi z}{a}\cdot sin\frac{m\pi z}{a}\cdot dz\\ I_{3}=\int_{0}^{a}cos\frac{l\pi z}{a}\cdot cos\frac{m\pi z}{a}\cdot dz \end{array}$$

The integrals exhibit orthogonal properties and ensure that:

(14) $$I_{2}=I_{3}=\{\begin{array}{c} \frac{1}{2}a,\;for\;l=m\\ \;0,\;for\;l\neq m \end{array}$$

Only when *l* and *m* are both odd or even numbers, the first integral *I*_1_ is zero. But due to the special structure of the *B* matrix, all terms that include *I*_1_ vanish (become zero). This means that the matrix ${\left(k^{lm}\right)}^{e}$ becomes a diagonal one, i.e., the non-zero values only exist in the diagonal area where *l* = *m*, which reduces the stiffness matrix to [14]:

(15) $${\left(k_{gk}^{ll}\right)}^{e}=\frac{1}{2}a\iint_{area}\left(B1_{g}^{l}{}^{T}DB1_{k}^{l}+B2_{g}^{l}{}^{T}DB2_{k}^{l}\right)dxdy\;l=1,\;2\ldots L$$

where *g*, *k* represent the element nodes, respectively. Area is the area of the element.

A typical term for the force vector becomes:

(16) $${\left(f^{l}\right)}^{e}={∭}_{vol}{\left(N^{l}\right)}^{T}{\left\{p\right\}}^{l}dxdydz$$

The global linear system is achieved by assembling the elemental stiffness matrix to the global domain:

(17) $$\left[\begin{array}{cccc} K^{11} & & & \\ & K^{22} & & \\ & & \ddots & \\ & & & K^{LL} \end{array}\right]\left\{\begin{array}{c} U^{1}\\ U^{2}\\ \vdots \\ U^{L} \end{array}\right\}+\left\{\begin{array}{c} F^{1}\\ F^{2}\\ \vdots \\ F^{L} \end{array}\right\}=0$$

The large system of equations can be divided into L separate problems:

(18) $$K^{ll}U^{l}+F^{l}=0$$

where *K* is the global stiffness matrix; *U* is the global displacement vector; *F* is the global loading vector.

For analysis of dynamic response in asphalt pavement, the time coordinates are introduced to the FE algorithm. The motion equation can be expressed based on Newton’s second law of motion:

(19) $$M\ddot{U}\left(t\right)+C\dot{U}\left(t\right)+KU\left(t\right)=F\left(t\right)$$

where U(t), $\dot{U}\left(t\right)$ and $\ddot{U}\left(t\right)$ are global displacement, velocity and acceleration vectors and U(0) = 0, $\dot{U}\left(0\right)$ = 0 and $\ddot{U}\left(0\right)$ = 0. *M*, *C* and *K* represent the global mass, damping and stiffness matrices, respectively. F(t) is the force vector.

The *M*, *C* and *K* are assembled by element mass, damping and stiffness matrices ${\left(M^{lm}\right)}^{e}$, ${\left(C^{lm}\right)}^{e}$ and ${\left(K^{lm}\right)}^{e}$, which are:

(20) $$\begin{array}{c} {\left(M^{lm}\right)}^{e}={∭}_{vol}{\left(N^{l}\right)}^{T}\rho N^{m}dxdydz\\ {\left(C^{lm}\right)}^{e}={∭}_{vol}{\left(N^{l}\right)}^{T}\mu N^{m}dxdydz\\ {\left(K^{lm}\right)}^{e}={∭}_{vol}{\left(B^{l}\right)}^{T}DB^{m}dxdydz \end{array}$$

where *ρ*, *μ* and *D* are the density, damping factor and elastic matrices, respectively.

As the same with static one, these matrices are diagonal and the global equations are as follows:

(21) $$\begin{array}{c} \left[\begin{array}{cccc} M^{11} & & & \\ & M^{22} & & \\ & & \ddots & \\ & & & M^{LL} \end{array}\right]\left\{\begin{array}{c} \ddot{U}{\left(t\right)}^{1}\\ \ddot{U}{\left(t\right)}^{2}\\ \vdots \\ \ddot{U}{\left(t\right)}^{L} \end{array}\right\}+\left[\begin{array}{cccc} C^{11} & & & \\ & C^{22} & & \\ & & \ddots & \\ & & & C^{LL} \end{array}\right]\left\{\begin{array}{c} \dot{U}{\left(t\right)}^{1}\\ \dot{U}{\left(t\right)}^{2}\\ \vdots \\ \dot{U}{\left(t\right)}^{L} \end{array}\right\}\\ +\left[\begin{array}{cccc} K^{11} & & & \\ & K^{22} & & \\ & & \ddots & \\ & & & K^{LL} \end{array}\right]\left\{\begin{array}{c} U{\left(t\right)}^{1}\\ U{\left(t\right)}^{2}\\ \vdots \\ U{\left(t\right)}^{L} \end{array}\right\}=\left\{\begin{array}{c} F{\left(t\right)}^{1}\\ F{\left(t\right)}^{2}\\ \vdots \\ F{\left(t\right)}^{L} \end{array}\right\} \end{array}$$

Thus, the large system also splits up into *L* separate problems:

(22) $$M^{ll}\ddot{U}{\left(t\right)}^{l}+C^{ll}\dot{U}{\left(t\right)}^{l}+K^{ll}U{\left(t\right)}^{l}=F{\left(t\right)}^{l}$$

According to Equations (16) and (21), the harmonics of the Fourier series are decoupled in the global equations, which is a benefit to the parallel calculation, and thus the computational time can be significantly reduced compared to a sequential solving procedure [26].

## 3. Time Discretization for Dynamic Analysis

A modal method or a direct integration method is usually used in FE analysis to undertake dynamic response analysis. No transformation to a special form is required in the direct integration method and the governing equation such as Equation (22) is solved step-by-step in the time domain.

One of the integration methods popularly used in the FE program for the solution of structural dynamic problems for both blast and seismic loading is called the Newmark method, which was proposed by Newmark in 1959 [27]. The Newmark method has been applied to the dynamic analysis of many engineering structures in the past several decades. A brief description of this method specialized for the SAFEM is provided here in two approaches: displacement based and acceleration based approach.

### 3.1. Displacement Based Approach

Within the time step (*t*, *t* + ∆*t*), the standard form of Newmark’s equations should be modified according to SAFEM as:

(23) $$\dot{U}{\left(t+\Delta t\right)}^{l}=\dot{U}{\left(t\right)}^{l}+\left[\left(1-\delta \right)\ddot{U}{\left(t\right)}^{l}+\delta \ddot{U}{\left(t+\Delta t\right)}^{l}\right]\Delta t$$

(24) $$U{\left(t+\Delta t\right)}^{l}=U{\left(t\right)}^{l}+\dot{U}{\left(t\right)}^{l}\Delta t+\left[\left(\frac{1}{2}-\alpha \right)\ddot{U}{\left(t\right)}^{l}+\alpha \ddot{U}{\left(t+\Delta t\right)}^{l}\right]\Delta t^{2}$$

where α and δ are algorithm parameters which are determined by the requirement of integral accuracy and stability. When δ = $\frac{1}{2}$, α = $\frac{1}{4}$ and δ = $\frac{1}{2}$, α = $\frac{1}{6}$ it becomes average acceleration method and linear acceleration method, respectively.

From Equation (24), one can derive the following equation:

(25) $$\begin{array}{c} \ddot{U}{\left(t+\Delta t\right)}^{l}=b_{1}\left(U{\left(t+\Delta t\right)}^{l}-U{\left(t\right)}^{l}\right)-b_{2}\dot{U}{\left(t\right)}^{l}-b_{3}\ddot{U}{\left(t\right)}^{l}\\ \mathrm{with}\;b_{1}=\frac{1}{\alpha \Delta t^{2}},\;b_{2}=\frac{1}{\alpha \Delta t},\;b_{3}=\frac{1}{2\alpha }-1 \end{array}$$

It is now substituted into Equation (22) to give the result:

(26) $$\begin{array}{c} \dot{U}{\left(t+\Delta t\right)}^{l}=b_{3}\left(U{\left(t+\Delta t\right)}^{l}-U{\left(t\right)}^{l}\right)+b_{4}\dot{U}{\left(t\right)}^{l}+b_{5}\ddot{U}{\left(t\right)}^{l}\\ \mathrm{with}\;b_{3}=\frac{\delta }{\alpha \Delta t},\;b_{4}=1-\frac{\delta }{\alpha },\;b_{5}=\Delta t-\frac{\delta \Delta t}{2\alpha } \end{array}$$

The linear dynamic equilibrium equation used in SAFEM is written in the form of Equation (22). The substitution of Equations (25) and (26) into Equation (22) allows the dynamic equilibrium of the system at time “t+Δt” to be written in terms of the unknown node displacements $U{\left(t+\Delta t\right)}^{l}$ for lth harmonic of Fourier series:

(27) $$\begin{array}{c} \bar{K^{ll}}U{\left(t+\Delta t\right)}^{l}=\bar{F{\left(t+\Delta t\right)}^{l}}\\ \mathrm{with}\;\bar{K^{ll}}=b_{1}M^{ll}+b_{3}C^{ll}+K^{ll},\;\bar{F{\left(t+\Delta t\right)}^{l}}=F{\left(t+\Delta t\right)}^{l}+M^{ll}(b_{1}U{\left(t\right)}^{l}+\\ b_{2}\dot{U}{\left(t\right)}^{l}+b_{3}\ddot{U}{\left(t\right)}^{l})+C^{ll}\left(b_{3}U{\left(t\right)}^{l}-b_{4}\dot{U}{\left(t\right)}^{l}-b_{5}\ddot{U}{\left(t\right)}^{l}\right) \end{array}$$

where $\bar{K^{ll}}$ is effective dynamic stiffness matrix and $\bar{F{\left(t+\Delta t\right)}^{l}}$ is effective load vector for lth harmonic of Fourier series.

The Newmark direct integration algorithm based on displacements is summarized as follows [28]: The calculation for the constants $b_{i}$ needs to be carried out only once. For linear systems, the effective dynamic stiffness matrix $\bar{K^{ll}}$ is formed and triangularized only once.

- Step 1: Initial Calculation
  (a) Form static stiffness matrix *K^ll^*, mass matrix *M^ll^* and damping matrix *C^ll^*.
  (b) Specify time step Δt and integration parameters α, δ.
  (c) Calculate integration constants $b_{i}$.
  (d) Form effective stiffness matrix $\bar{K^{ll}}$.
- Step 2: For Each Time Step t=0,Δt,2Δt,3Δt⋯
  (a) Specify initial conditions $U{\left(0\right)}^{l}$, $\dot{U}{\left(0\right)}^{l}$ and $\ddot{U}{\left(0\right)}^{l}$.
  (b) Calculate effective load vector $\bar{F{\left(t+\Delta t\right)}^{l}}=\bar{F{\left(\Delta t\right)}^{l}}$.
  (c) Solve for node displacement vector at time t according to Equation (27).
  (d) Calculate node velocities and accelerations at time t according to Equations (25) and (26).
  (e) Go to Step 2 (*b*) with t= t+Δt.

### 3.2. Acceleration-Based Approach

The unknown node accelerations $\ddot{U}{\left(t+\Delta t\right)}^{l}$ for lth harmonic of Fourier series can be derived from substitution of Equations (23) and (24) into Equation (22) directly:

(28) $$\begin{array}{c} \bar{K^{ll}}\ddot{U}{\left(t+\Delta t\right)}^{l}=\bar{F{\left(t+\Delta t\right)}^{l}}\\ \mathrm{with}\;\bar{K^{ll}}=M^{ll}+\delta \Delta tC^{ll}+\alpha \Delta t^{2}K^{ll},\Delta \bar{F{\left(t+\Delta t\right)}^{l}}=F{\left(t+\Delta t\right)}^{l}-C^{ll}[\dot{U}{\left(t\right)}^{l}+(1-\\ \delta )\Delta t\ddot{U}{\left(t\right)}^{l}]-K^{ll}\left[U{\left(t\right)}^{l}+\Delta t\dot{U}{\left(t\right)}^{l}+\left(\frac{1}{2}-\alpha \right)\Delta t^{2}\ddot{U}{\left(t\right)}^{l}\right] \end{array}$$

In this approach, the incremental equations of motion are solved for incremental acceleration first, and the incremental velocity and displacement are calculated from this incremental acceleration according to Equations (23) and (24). The numerical integration algorithm based on this scheme approach is similar to that based on displacements.

In the code of SAFEM, the acceleration based approach with the average acceleration method is adopted.

## 4. Analytical Verification of SAFEM in Dynamic Analyses

The accuracy of dynamic analyses under moving loads using SAFEM was verified by comparing the results with the data derived from ABAQUS (Abaqus 6.14-1, Johnston, RI, USA). The responses from both models were evaluated with the pavement type in Table 1, which is widely used in Germany. The thicknesses of all layers, excluding the subgrade, were derived from the guideline RStO 12 [29]. The thickness of the subgrade was defined as 2000 mm; setting such a large value aims to minimize the influence of the boundary condition on the results. The length and width of all layers were set to 6000 mm for a similar reason. Homogeneous E-moduli in each layer were defined according to the guideline RDO Asphalt 09 [30] for assumed pavement surface temperatures of 27.5 *C* which is the typical temperature in the summer in Germany.

**Figure 2:**
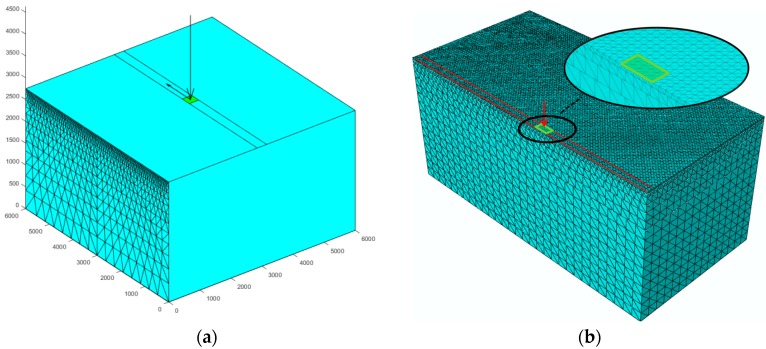
Mesh automatically generated from (**a**) SAFEM and (**b**) ABAQUS.

The 2D mesh generated from SAFEM was as shown in Figure 2a. A 3D 10-node quadratic tetrahedron element was applied in ABAQUS due to its three-dimensional discretization. In order to save computational time of the analysis, only a half-symmetrical model was created in ABAQUS, whereas a full size model was created in SAFEM. The model with the mesh algorithm in ABAQUS is illustrated in Figure 2b. Due to the different dimensions of both models, the mesh algorithms are different, while the general mesh regulation is the same in both models, i.e., the element size is gradually increased from the loading area to periphery.

The load in SAFEM and ABAQUS was assumed as a square load and its side length is 300 mm and the speed is 52 km/h. The uniformly distributed contact pressure was 0.7 MPa. The load path traverses the center of the pavement, as shown in Figure 2. The element size in ABAQUS is 60 mm in the direction of traffic and 50 mm in the transverse direction, as shown in Figure 2b. The load moved forward one element in each increment by default; therefore there were 100 increments in both analyses. It should be mentioned that currently the loading mode of moving loads in ABAQUS is not offered in its graphical user interface (GUI) [31], i.e., the user has to write a subroutine to realize this function which is difficult for common pavement engineers.

The bottom nodes of the mesh representing the subgrade in both models were fixed in all directions. On both edges (*z* = 0 and *z* = *a*), the displacements are restricted in the *x*- and *y*-directions due to the theory of SAFEM. The equivalent boundary conditions were also used in the ABAQUS model. The three asphalt layers were totally bound; the two contact layers among the asphalt base course, road base course, sub-base and subgrade were defined as being partially bound.

**Figure 3:**
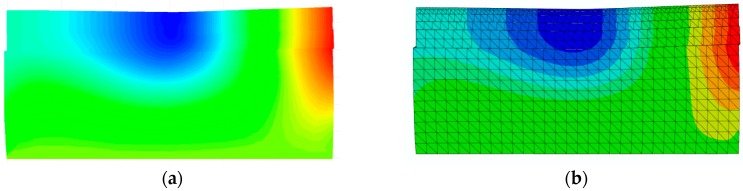
The computational vertical displacement from (**a**) SAFEM and (**b**) ABAQUS with a scale factor of 500 in dynamic analysis under moving loads.

The computational vertical displacements obtained from both models when the load is at the center of the pavement (the 50th increment) are illustrated in a Moiré pattern, as shown in Figure 3. The cross-section is through the centroid of the full size pavement model and along the traffic direction. It can be seen that the distribution of the displacement, magnitude and the deformation shapes from both FE programs are in good agreement with one other.

**Figure 4:**
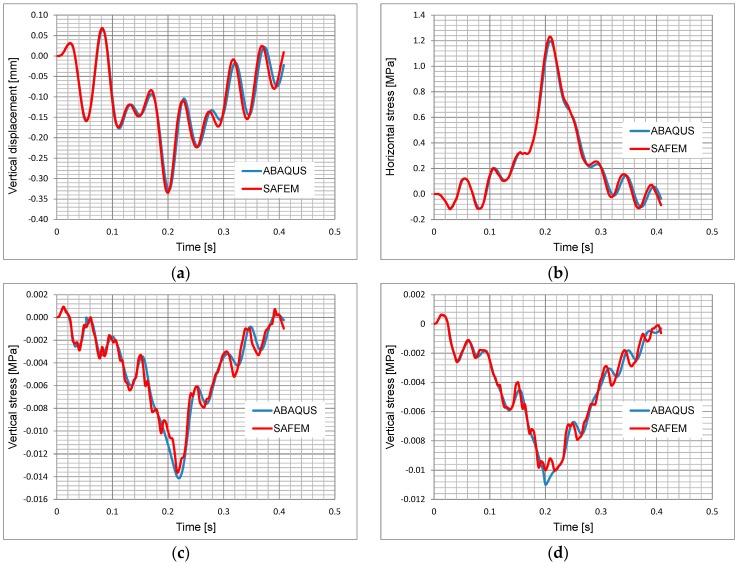
Comparison of the results between ABAQUS and SAFEM. (**a**) Vertical displacement at the top of the surface course; (**b**) Horizontal stress at the bottom of the asphalt base course; (**c**) Vertical stress at the top of the sub-base course; (**d**) Vertical stress at the top of the subgrade.

The computational results from four response points in the loading history are shown in Figure 4.

The corresponding results from Figure 4 at a loading time of 0.2 s are listed in Table 2. As seen in Figure 4 and Table 2, it can be stated that the results obtained from both programs have a high correlation considering that their mesh algorithms are totally different due to the different dimensions of discretization.

Benefit from the use of a Fourier series in the transverse direction the SAFEM needs significantly fewer elements and nodes compared with ABAQUS; this results in a far shorter computational time for the SAFEM as compared to ABAQUS. A computer with the configuration of an Intel Core Duo 3.4 GHz and 32 GB RAM was used to run the two FE analyses. Generally, the computational time required by the half-symmetrical 3D model in ABAQUS is about 281 min, whereas the full-size pavement model in SAFEM requires 10 min, which is only 3.6% of that used by ABAQUS, as shown in Table 3.

## 5. Experimental Verification of SAFEM in Dynamic Analyses

**Figure 5:**
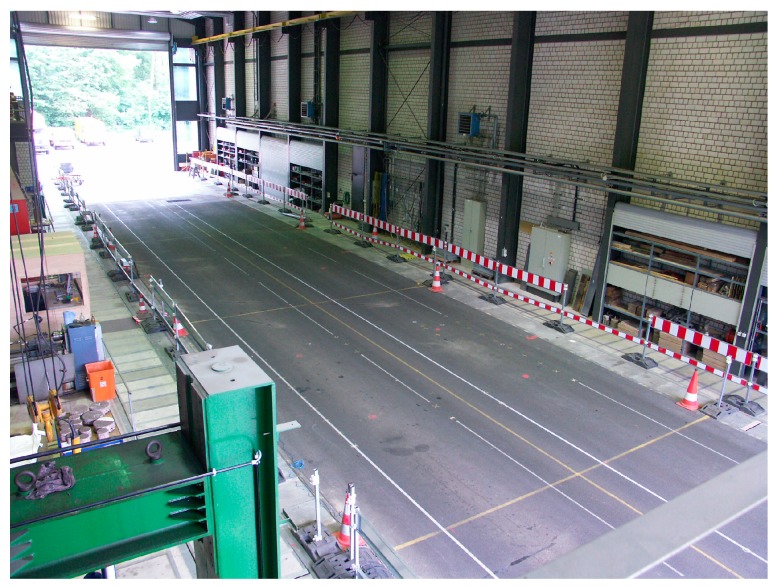
The test track in German Federal Highway Research Institute (BASt).

**Figure 6:**
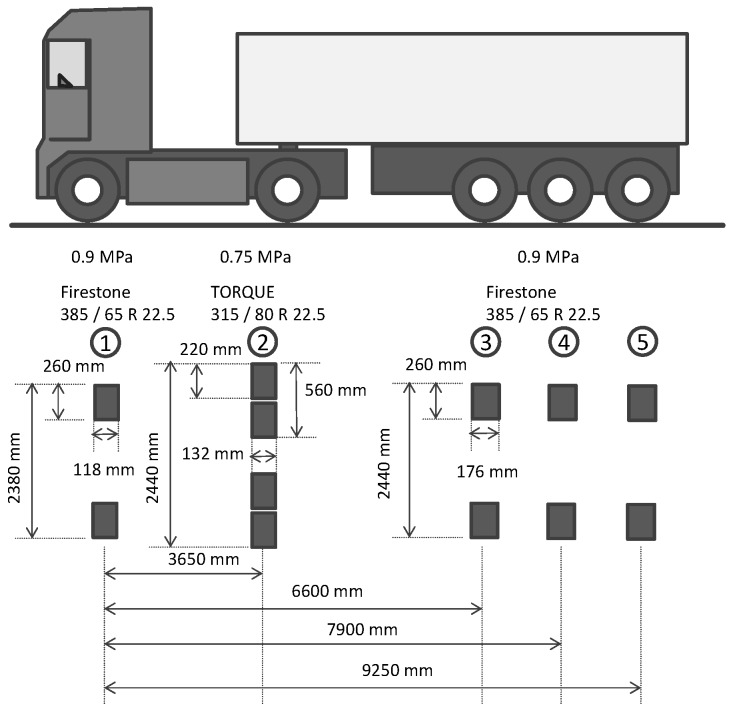
Geometrical and loading data of the truck.

A test track in the German Federal Highway Research Institute (BASt) was used in this study to experimentally verify the SAFEM, as shown in Figure 5. The test track is comprised of five layers, which are asphalt surface course, asphalt binder course, asphalt base course, frost protection layer and subgrade with the thickness of 40, 50, 130, 680, and 1440 mm, respectively. Strain gauges were placed at the bottom of the asphalt base course during the test track construction, which is to measure strains along the traffic direction for the verification. A passing truck with speed of 30 km/h was used to apply the moving loads in the experiment. The geometry and the distribution of the loads are illustrated in Figure 6. The left wheels of five axles were considered in the verification in order to simplify the model and thus reduce the computational time. The specimens were drilled from the test track to obtain the material properties and then the material parameters were used in SAFEM. More details can be found from [19,32].

When the second wheel load was exactly above the locations where the sensors were embedded the computational strains from SAFEM were compared with the field measurement, as shown in Table 4. The computational strain at the bottom of the asphalt base course is higher than the measured value with a difference of 5.88%. The measured values can be accepted in a range of 20% due to the uncertainties and fluctuations [19], the computational strains are therefore in a quite good agreement with the measurement.

## 6. Summary and Conclusions

This paper proposes to use the SAFEM for predicting the asphalt pavement dynamic responses under moving loads. The accuracy of the program is analytically and experimentally verified by comparison with ABAQUS and field measurements, respectively. The results predicted by SAFEM and ABAQUS are generally consistent with each other. Furthermore, the efficiency of the SAFEM is much higher than that of the ABAQUS. The computational strain derived from SAFEM at the critical point is quite close to the field measurement, which further proves the accuracy of the developed program.

In conclusion, the SAFEM has potential to reliably analyze the dynamic response of asphalt pavement under moving loads. For further investigation, the SAFEM allows the application of various material properties, such as viscoelasticity for asphalt and nonlinear elasticity for the sub-base of the pavement. With these improvements, the SAFEM should be more appropriate to predict the impact of the moving load on the asphalt pavement.

## Figures and Tables

**Table 1:** Geometrical data and material properties of the pavement in dynamic analysis under moving loads.

Layer	Thickness (mm)	E (MPa)	µ	Density (t/mm^3^)
Surface course	40	22,690	0.35	2.377 × 10^−9^
Binder course	80	27,283	0.35	2.448 × 10^−9^
Asphalt base course	140	17,853	0.35	2.301 × 10^−9^
Road base course	150	10,000	0.25	2.400 × 10^−9^
Sub-base	340	100	0.49	2.400 × 10^−9^
Subgrade	2000	45	0.49	2.400 × 10^−9^

**Table 2:** Comparison between ABAQUS and SAFEM regarding the computational results at critical points when the loading time is 0.2 s.

Result	SAFEM	ABAQUS	Difference
Vertical displacement (mm) at the top of the surface course	−0.335	−0.327	2.44%
Horizontal stress (MPa) at the bottom of the asphalt base course	0.974	0.912	6.79%
Vertical stress (MPa) at the top of the sub-base course	−0.0104	−0.0112	−7.14%
Vertical stress (MPa) at the top of the subgrade	−0.0100	−0.0109	−8.26%

**Table 3:** Comparison of the efficiency between SAFEM and ABAQUS.

	SAFEM	ABAQUS
Elements	1144	127,095
Nodes	2431	218,333
Computational time	10 min	281 min

**Table 4:** Comparison of the strains derived from measurement and SAFEM.

	Measurement	SAFEM	Difference
Strain along the traffic direction at the bottom of the asphalt base course (10^−6^)	81.5	86.3	5.88%
